# The Role of Matrix Metalloproteinases in the Epithelial-Mesenchymal Transition of Hepatocellular Carcinoma

**DOI:** 10.1155/2019/9423907

**Published:** 2019-11-26

**Authors:** Cristian Scheau, Ioana Anca Badarau, Raluca Costache, Constantin Caruntu, Gratiela Livia Mihai, Andreea Cristiana Didilescu, Carolina Constantin, Monica Neagu

**Affiliations:** ^1^Department of Physiology, Carol Davila University of Medicine and Pharmacy, Bucharest 050474, Romania; ^2^Gastroenterology and Internal Medicine Clinic, Carol Davila University Central Emergency Military Hospital, Carol Davila University of Medicine and Pharmacy, Bucharest 050474, Romania; ^3^Department of Dermatology, Prof. N.C. Paulescu National Institute of Diabetes, Nutrition and Metabolic Diseases, Bucharest 011233, Romania; ^4^Department of Embryology, Faculty of Dental Medicine, Carol Davila University of Medicine and Pharmacy, Bucharest 050474, Romania; ^5^Immunology Department, Victor Babes National Institute of Pathology, Bucharest 050096, Romania; ^6^Department of Pathology, Colentina University Hospital, Bucharest 020125, Romania; ^7^Faculty of Biology, University of Bucharest, Bucharest 76201, Romania

## Abstract

The epithelial-mesenchymal transition (EMT) is a transformation process mandatory for the local and distant progression of many malignant tumors, including hepatocellular carcinoma (HCC). Matrix metalloproteinases (MMPs) play significant roles in cellular regeneration, programmed death, angiogenesis, and many other essential tissular functions, involved in the normal development and also in pathological processes, such as the EMT. This paper reviews the roles of MMPs in the EMT involved in HCC invasion, as well as the ancillary roles that MMP cross-activation and tissue inhibitors play in modulating this process. While gelatinases MMP-2 and MMP-9 are the MMPs commonly cited in the EMT of HCC, MMPs belonging to other classes have been proven to be involved in this process, favoring not only invasion and metastasis (MMP-1, MMP-3, MMP-7, MMP-10, MMP-11, MMP-13, MMP-14, MMP-16, MMP-26, and MMP-28) but also angiogenesis (MMP-8 and MMP-10). There is also data suggesting that other MMPs with a suspected or demonstrated role in the EMT of other cancers may also have some degree of involvement in HCC. The auto- and cross-activation of MMPs may complicate this issue, as pinpointing the extent of implication of each MMP may be extremely difficult. The homeostasis between MMPs and their tissue inhibitors is essential in preventing tumor progression, and the disturbance of this stability is another entailed factor in the EMT of HCC, which is addressed herein.

## 1. Introduction

Hepatocellular carcinoma (HCC) is one of the leading causes of death worldwide and develops in a context of long-term liver injury, inflammation, and regeneration [1]. With a mortality of 9.1% worldwide, HCC is the fifth most common cancer and is considered a significant global health burden, by itself and through its potential unnoticeable or overt complications [2–4]. With recent progress, diagnosis hindrances of rare or atypical forms of HCC have been surmounted, and novel therapies appear promising in complementing the available resources for managing this disease [5–7]. However, a better understanding of the underlying pathological mechanisms in the development of HCC may uncover more efficient ways to limit tumor growth and dissemination.

Epithelial-mesenchymal transition (EMT) is defined as a transformation process, in which epithelial cell features are lost in favor of adopting mesenchymal traits; the process usually implies loss of the apicobasal cell polarity, through intracellular adhesion alteration. EMT is considered essential for oncogenesis, enabling tumors to acquire aggressive features such as invasiveness and the ability to metastasize [8].

Matrix metalloproteinases (MMPs) are a family of zinc-dependent endoproteases responsible for degrading the extracellular matrix (ECM) by breaking down various proteins in its structure. MMPs promote a wide spectrum of processes, including cell proliferation and migration, and could play a role in cell apoptosis, angiogenesis, tissue regeneration, and immune response [9]. In malignancies, such as HCC, MMPs function within the tumor microenvironment to induce changes during EMT and help to facilitate EMT via invasion and metastasis behaviors [10].

MMPs seem to play important roles, as the members of this family have various implications in the complex pathogenesis of EMT in HCC. This paper is aimed at thoroughly presenting their functions in this process.

## 2. Matrix Metalloproteinases

### 2.1. General Description

MMPs belong to metzincins, a family of Zn^2+^-dependent, Ca^2+^-containing endoproteases comprising of 24 members in mammals (23 in humans). MMPs are produced as zymogens (pro-MMPs) that are activated by other enzymes or free radicals through the cysteine switch mechanism [11].

Metalloproteinases are named incrementally, starting with MMP-1 and ending with MMP-28, but not including MMP-4, MMP-5, MMP-6, and MMP-22, since these enzymes were discovered simultaneously by different research teams. MMP-18 was identified in Xenopus with no known human orthologue [12]. Based on the target substrate and chemical structure, MMPs are split into several groups: collagenases, gelatinases, stromelysins, matrilysins, membrane-type MMPs, and other nonclassified MMPs [13].

MMPs promote the degradation of various ECM proteins, leading to architectural changes in the cell and tissue environments. Different MMPs have different efficacies in breaking down various proteins. Some of the substrates targeted by MMPs include collagen, gelatin, aggrecan, entactin, fibronectin, laminin, tenascin, and vitronectin. MMPs can also degrade myelin basic protein and casein [14, 15].

Cytokines, chemokines, and various receptors may also be targeted by various MMPs. As such, MMPs not only break down ECM components but also are involved in regulating intra- and intercellular signaling pathways by proteolysis [16].

MMP-1, MMP-2, MMP-3, MMP-11, and MMP-13 are among the MMPs constitutively expressed in normal livers, while the others may appear in various pathological processes, such as acute or chronic liver injury [17].

There are several levels of regulation for MMPs: gene transcription, secretion compartmentalization, proenzyme activation, endocytosis, and inhibition of enzyme activity [15, 16, 18].

The inhibition of MMPs is performed by either endogenous or exogenous inhibitors. Tissue inhibitors of metalloproteinases (TIMPs) may inhibit more than one metalloproteinase and are widely distributed in the human body [19]. Either absolute or relative changes in MMP or TIMP levels can trigger important changes in specific MMP activity [15]. TIMP-1 and TIMP-2 can block MMP effects in promoting tumor cell proliferation and migration and can also inhibit angiogenesis and apoptosis. Conversely, apoptosis is induced by TIMP-3 through TNF-*α* receptor stabilization and by TIMP-4 via overexpression and through different mechanisms, depending on the region involved [20].

### 2.2. MMP Interaction

Once activated, MMPs subsequently activate other MMPs that are in an inactive zymogen form (pro-MMPs). This may lead to a cascade of activation, and this network of interaction between MMPs may potentiate their effects in the EMT.

MMP-3 and MMP-10 activate MMP-1, MMP-7, MMP-8, and MMP-9, enhancing ECM degradation [21]. MMP-14 activates both MMP-2 and MMP-13, in the presence of TIMP-2, with a demonstrated effect in tumor invasion and metastasis, by promoting cell migration [22].

MMP-15, MMP-16, and MMP-24 also activate MMP-2 on the cell surface, affecting the ECM integrity [23]. Other interactions between MMPs, such as activation of MMP-13 by MMP-15, MMP-2, and MMP-3, as well as activation of MMP-9 by MMP-2 and MMP-13 and activation of MMP-2 by MMP-1, MMP-17, MMP-7, MMP-13, and MMP-25, were also described [24].

MMP-1 was also shown to be activated by MMP-7, and MMP-13 may also be activated by MMP-10, while MMP-14 and MMP-26 are capable of autoactivation [22, 25]. Activated MMP-7 can also determine the activation of MMP-9 and MMP-13, while MMP-12 could activate MMP-2 and MMP-3 [26].

This intricate web of MMP cross-activation is able to completely cleave the ECM if the strict and multilevel regulation of MMPs is overwhelmed [26]. Also, the multiple interactions make it difficult to accurately determine the individual role of each MMP in an *in vivo* setting of HCC cells going through EMT.

## 3. EMT and HCC

EMT is a biological process defined as a rigorously programmed shift from epithelial to mesenchymal cell features that plays a substantial role in embryogenesis and organ development and also tissue repair and regeneration, as well as tumor invasion and metastasis [27]. EMT is triggered and sustained by multiple molecular processes, which, in some cases, may be used as biomarkers. Some of the genomic processes include activation of transcription factors and alterations of microRNAs, while nongenomic processes involve release of MMPs, cytoskeletal reconfiguration, and expression of specific proteins on the cell surface [28].

The main features of the EMT include the downregulation of epithelial markers, such as E-cadherin, beta-catenin, tight junction protein-1, laminin, and cytokeratin, and the upregulation of mesenchymal markers such as N-cadherin, vimentin, and *α*-smooth muscle actin (*α*-SMA). A number of transcription factors are involved in the EMT of HCC, including Snail, Twist, and zinc finger E-box binding protein 1 (ZEB1), and their presence is associated with a poor prognosis [29–31]. These EMT transcription factors activate multiple cellular signaling pathways and molecules, such as Akt, MAPK, STAT3, transforming growth factor beta (TGF-*β*), *β*-catenin, Wnt, Ras, Notch, nuclear factor *κ*B (NF-*κ*B), tumor necrosis factor alpha (TNF-*α*), and hypoxia-inducible factor-1 alpha (HIF-1*α*) [31].

The involvement of several MMPs in tumor progression through EMT was demonstrated in various digestive (gastric, pancreatic, and colorectal) and nondigestive (lung, ovarian, mammary, and prostate) cancers, with similarities to HCC, but entailing tissue-specific pathogeneses [32–38].

Some effects of the EMT in HCC, such as tumor invasion, were demonstrated on HepG2 and Huh-7 cell lines; for instance, Snail was reported to present a strong effect in increasing invasion, correlated with cell dedifferentiation. The MMP gene family is upregulated by Snail expression in HepG2 cells in candidate genes relating to tumor migration; therefore, MMPs may play an important part in the EMT of HCC [39].

## 4. MMPs with a Demonstrated Role in the EMT of Invasive HCC

**Figure 1:**
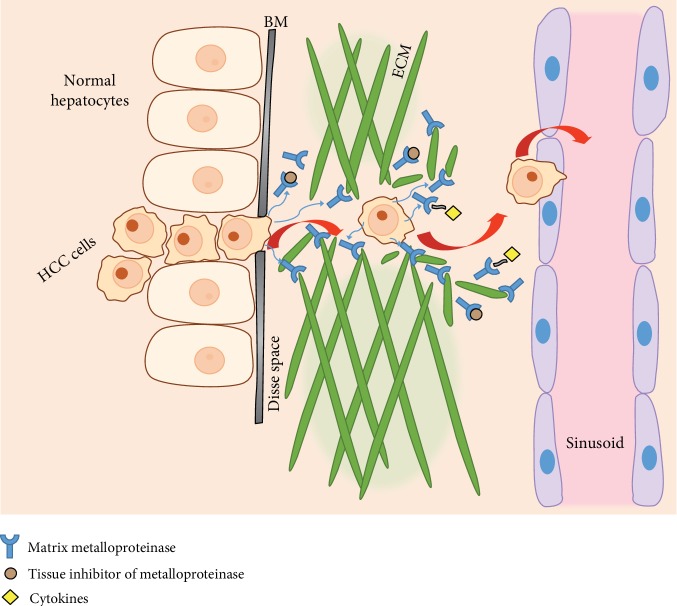
Schematic representation of the epithelial-mesenchymal transition of hepatocellular carcinoma (HCC) cells. Matrix metalloproteinases (MMPs) break down the extracellular matrix (ECM) and activate various cytokines facilitating the local invasion of the HCC cells through the basement membrane (BM) and Disse space, favoring their advance towards the liver sinusoids. Tissue inhibitors of metalloproteinases block the activity of some MMPs, balancing the process.

Degradation of the basement membrane and subsequently of the ECM is critical for invasion and metastasis, and these complications are major factors of poor prognosis in HCC patients [40]. MMPs are key factors in providing the invasive and metastatic traits of malignant tumor cells by enabling their infiltration and migration in the process of EMT. Tumor cell migration depends on the increased release and activation of MMPs, as well as their cell membrane expression, leading to a breakdown of the ECM and favoring infiltration [41] (see Figure 1).

*MMP-1* is also named collagenase-1, due to the fact that it promotes degradation of the interstitial collagens and is normally regulated by TIMP-1. Overexpression of both MMP-1 and TIMP-1 is associated with an elevated invasive and migratory capacity of the HCC cells, most likely by ECM degradation in the process of EMT [42]. Overexpression of miR-526b is associated with increased cell proliferation, venous infiltration, and a poor prognosis, and MMP-1 is recognized as a direct target of miR-526b [43].

*MMP-2* is a collagenase that represents the main proteolytic enzyme among MMPs and is a major promoter of tumor cell invasion and metastasis through breaking down of the basement membrane and favoring the local and distant infiltration of tumor cells [44]. MMP-2 is not normally found in liver cells but is expressed in HCC cells, especially in the fibrolamellar variant [45]. In the EMT of HCC, MMP-2 seems to be linked to HIF-1*α*, a known enhancer of tumor invasion and metastasis, which downregulates E-cadherin and upregulates MMP-2 [46].

*MMP-3*, or stromelysin-1, can degrade a variety of ECM substrates, including collagens, laminins, fibronectin, osteopontin, and proteoglycans, while also demonstrating a proteolytic activity on cell surface protein ectodomains. Hepatocyte growth factor (HGF) stimulates MMP-3 to initiate and maintain the EMT of HCC, favoring the invasion of ECM by liver cancer cells [47]. The role of MMP-3 in the invasiveness of HCC can be demonstrated by its *in vivo* expression, while HGF-induced invasion can be demonstrated by using an antibody to MMP-3, which blocks the invasion [48]. Features of invasion and migration in HCC can be stimulated by some cytokines, such as IL-1*β*, TNF-*α*, and interferon gamma, which can induce a significant MMP-3 mRNA production that in normal circumstances is at low levels [49].

*MMP-7* is also known as matrilysin, and it cleaves many protein components of the ECM, including collagen, entactin, osteopontin, fibronectin, laminin, elastin, and proteoglycans, and also pro-MMP-2 and pro-MMP-9, as well as other proteins [50]. MMP-7 was found to function as a prometastatic factor by promoting the migratory and invasive ability of cancer cells, and overexpression of MMP-7 was found in HCC specimens and cells, favoring EMT. MMP-7 is a direct target of miR-489 in HCC, and miR-489 inhibits the migration and invasion of HCC. The underexpression of miR-489 facilitates tumor migration that plays a role in HCC progression, via targeting MMP-7 [51].

*MMP-8*, or collagenase-2, plays a role in cell proliferation and migration, as well as in angiogenesis, through the development of capillary-like network structures [52]. Upregulation of MMP-8 and TGF-*β*1 activates the PI3K/Akt/Rac1 pathway, altering the EMT phenotype, inducing HCC invasion and migration [53]. The use of apigenin in HCC Huh-7 cells inhibits the migration capabilities of tumor cells through downregulation of vimentin, type I collagen, VEGF, and MMP-8, thus regulating angiogenesis and migration and promoting EMT [54].

Alongside MMP-2, *MMP-9*, also known as gelatinase B, is one of the most studied MMPs in the pathogenesis of EMT in HCC. MMP-9 degrades the ECM, activates IL-1*β*, and cleaves several chemokines [55]. MMP-9 seems to play a major role in tumor angiogenesis, through its critical intervention in the regulation of growth plate angiogenesis and recruitment of endothelial stem cells [56]. Overexpression of MMP-9 in HCC leads to a higher TNM stage through an increase of lymph node invasion as well as promoting metastasis and also to poor differentiation and an overall poor prognosis [57]. MMP-9 is considered a consistent progression marker alongside extracellular matrix protein 2, related to invasion and metastasis, and they represent targets to as many as 285 consistently downregulated and 149 upregulated genes appearing in the EMT of HCC [58].

*MMP-10*, or stromelysin-2, is mainly found in epithelial cells and is involved in tumor cell invasion and metastasis by targeting several pro-MMPs, as well as breaking down ECM components such as collagen, gelatin, elastin, fibronectin, proteoglycans, and laminin [59]. MMP-10 contributes to HCC development, participating in tumor angiogenesis, growth, and lung dissemination, induced by hypoxia, an increased CXCR4 expression, stromal-derived factor-1, and increased C-Jun transcriptional activity, resulting in the EMT of HCC cells [60–62].

*MMP-11*, also known as stromelysin-3, has a relatively limited substrate, by only cleaving the insulin-like growth factor-binding protein-1, the laminin receptor, and the native alpha3 chain of collagen VI. Nevertheless, MMP-11 overexpression is a factor of poor prognosis in various human carcinomas. Interestingly, this proteinase is not expressed in malignant cells themselves but is secreted by adjacent mesenchymal cells that do not present specific malignant features [63]. One of the novel biomarkers of tumor aggressiveness and potential targets for HCC treatment is miR-125a, which decreases the EMT activity by downregulating MMP-11 and VEGF, *in vitro* and *in vivo*, resulting in an inhibition of HCC invasion and migration [64].

*MMP-13* (collagenase-3) is activated by TGF-*β* and is important in HCC invasion and metastasis. TGF-*β* seems to only be involved in invasive HCC types, and the stimulating effects on MMP-13 expression are correlated with a feedback repression of miR-127 [65]. High levels of MMP-13 and of gelatinases are responsible for the degradation of the basement membrane, favoring EMT [66].

*MMP-14* is a membrane-type MMP that plays an important role in cancer metastasis by degrading the ECM, increasing the secretion of pro-MMP-2 and pro-MMP-9, and interacting with TIMP-2. The increased expression of MMP-14 seems to be correlated with high rates of portal vein invasion, intrahepatic metastasis, and recurrence in HCC [67]. Pravastatin reduces the rates of local invasion and distant metastasis in HCC by decreasing the expression of MMP-14 required for MMP-2 activation [68].

*MMP-16* is a membrane-type MMP localized on the surface of fibroblasts, capable of degrading various ECM components, including collagen, and is an activator of MMP-2 [69]. MMP-16 induces EMT in HCC, promoting cancer cell invasion and metastases; silencing MMP-16 expression hinders the EMT process by increasing the expression of epithelial cell marker E-cadherin while repressing mesenchymal markers vimentin and N-cadherin [40].

*MMP-26*, or matrilysin-2, breaks down several EMC components and activates MMP-9 through cleavage. MMP-26 may be activated in HCC Huh7 cells when stimulated by fibroblast growth factors that increase tumor proliferation and migration, with the involvement of the extracellular signal-regulated kinase (ERK) and NF-*κ*B pathways [70]. Also, tumor formation in distal organs was detected in mice that received MMP-26+CXCR4+HepG2 HCC cells, suggesting that MMP-26 plays a role in the EMT of HCC [71].

*MMP-28*, also known as epilysin, is the most recently identified MMP and degrades casein. MMP-28 promotes and maintains EMT through activation of TGF-*β* signaling and upregulating Snail transcription factor [72]. Elevated levels of MMP-28 in HCC are correlated with a poor prognosis, due to higher TNM stage and increased rates of portal vein invasion and metastasis, the latter apparently depending on Notch3 signals [73].

The MMPs with demonstrated involvement in the EMT of HCC are presented in Table 1, alongside their MMP activators.

## 5. Other MMPs with Possible Involvement in Tumor Pathogenesis

*MMP-12* degrades elastin, thus earning its alternate name: macrophage elastase. MMP-12 expression affects overall survival time of patients with HCC who underwent curative resection but does not seem to be involved in HCC invasiveness or metastasis [75].

*MMP-15* is classified into the membrane-type MMPs that are important for pericellular proteolysis, and the expression level of MMP-15 is associated with tumor growth of human fibrosarcoma and gastric cancer cells as well as tumor progression and intratumoral angiogenesis in non-small-cell lung cancer [74, 76, 77]. However, in regard to MMP-15 involvement in HCC, only indirect and nondefinitive data is available to date [77].

*MMP-17* is a relatively newly discovered membrane-type MMP that is glycosylphosphatidylinositol- (GPI-) anchored, but little information is available in regard to its physiological roles [78]. No involvement of MMP-17 in the EMT of HCC was found in the literature, but it was implicated in breast cancer progression, apparently by facilitating *in vivo* and *in vitro* breast cancer cell proliferation through outside-in EGFR signaling, but without acting as a protease [79].

*MMP-19*, also known as stromelysin-4, could be involved in processes such as neovascularization and angiogenesis or lymphocyte extravasation, but its role in cancer evolution is unclear [80].

*MMP-20*, or enamelysin, is a tooth-specific MMP, which under normal conditions is only associated with ameloblasts and odontoblasts but was recently identified in colon, breast, and lung cancers [81]. HCC cells with increased serine protease inhibitor Kazal-type- (SPINK-) 6 expression associate a significant downregulation of MMP-20, as well as MMP-9, suggesting that MMP-20 may also play a role in ECM degradation and tumor cell invasion and migration [82].

*MMP-21* is involved in establishing left-right asymmetry by cleaving specific targets at the embryonic node and possibly activating latent TGF-*β* factors [83]. The involvement of MMP-21 in cell adhesion as well as in cell migration is made possible by the vitronectin-like domain in the catalytic site [84]. The increased expression of MMP-21 is correlated with a poor prognosis due to the higher TNM stage, tumor invasion, and metastasis in other types of malignancies, such as gastric cancer and colorectal cancer [85]. However, implication in HCC is undetermined as of yet.

*MMP-23* may possess a novel mechanism for cellular localization, due to a lack of C-terminal transmembrane domain or GPI anchor found in the membrane-type MMPs [86]. MMP-23 is upregulated in a Mdr2-knockout model of chronic inflammation-mediated HCC, possibly playing a still unclear role in the hepatocarcinogenesis process occurring in long-term liver inflammation [87].

*MMP-24* is a membrane-type MMP that activates MMP-2 by cleavage and was identified as a biomarker of lung and gastric adenocarcinoma progression and metastasis [88]. MMP-24 is expressed after partial hepatectomy, but no studies were found citing its role in the EMT of HCC [17].

*MMP-25* is another GPI-anchored membrane-type MMP that is expressed in several human cancers, including brain, colon, urothelial, and prostate cancers [89]. It was suggested that MMP-25 may be important for tumor cell invasion because elevated levels are identified in the tumor progression process of invasive colon cancer [90]. Also, MMP-25 reduces the levels of alpha-1 proteinase inhibitor, stimulating the ECM degradation and the subsequent tumor invasion and migration [91].

*MMP-27* has a unique C-terminal extension which does not lead to stable membrane insertion, favoring its retention in the endoplasmic reticulum [92]. MMP-27 was initially speculated as a marker of poor prognosis in breast cancer patients, but later studies did not confirm these findings [93]. Enhanced melanoma progression is often observed in patients that present mutations in the genes coding MMP-8 and MMP-27 [94]. The effect of MMP-27 on HCC invasiveness is yet to be determined.

## 6. TIMP Contribution

The balance between matrix metalloproteinases and TIMPs seems to be a key factor in maintaining a normal configuration of the ECM and of the basement membrane, as well as preventing tumor cell invasion and migration [95]. Each human TIMP may inhibit several MMPs with various affinities, but noninhibitory interactions may also occur between a series of MMP-TIMP couples, such as coactivation of pro-MMP-2 by TIMP-2 and activation protection of pro-MMP-9 by TIMP-1 [96].

*TIMP-1* overexpression facilitates the EMT of HCC cells, through functions that are independent of MMPs, such as modulating apoptosis, mitogenic activity, and cellular proliferation and morphology [97]. TIMP-1 can be used as a marker of lung metastasis in HCC, due to the fact that transcripts for TIMPs were clearly demonstrated in the metastatic HCC nodules in the lung [98]. Also, TIMP-1 initiates the transformation from liver fibroblasts to carcinoma-associated fibroblasts in the tumor milieu of HCC in progression [99].

*TIMP-2* serum and tissue concentrations are lower in HCC patients with metastasis and are higher in those without; furthermore, patients with high levels of TIMP-2 have higher survival rates than those with low levels [100]. Besides the aforementioned decreases in MMP-14 and, subsequently, MMP-2 levels, statins also trigger a decline in the expression of TIMP-2 and also TIMP-1, restoring the MMP-TIMP balance and reducing the progression and metastatic rates of HCC in a preclinical model [68].

*TIMP-3* may have a role in decreasing the aggressiveness of HCC, by inhibiting portal vein invasion and lymph node metastasis, probably by suppressing tumorigenesis and angiogenesis by interacting with integrin *α*7 and angiotensin II type 2 receptor [101]. However, TIMP-3 was found to be associated with tumor progression and negative clinical outcome in squamous cell carcinomas of the head and neck, so its definitive role in EMT remains to be established [102].

Exogenous TIMPs, such as fucoidan, maintain the ECM homeostasis by increasing TIMP-1 and decreasing MMP-2 and MMP-9 through downregulation of TGF-*β* signaling, implicitly decreasing the aggressiveness of HCC by preventing the EMT [103].

TIMPs play complex roles in balancing the activated MMPs and their roles in various processes, such as ECM degradation, angiogenesis, and tumor invasion and migration, but some of the effects of TIMPs are MMP-independent and have been demonstrated to favor HCC progression in some cases [104]. Serum concentrations of TIMPs, as well as some MMP/TIMP ratios, seem to correlate with the prognosis and overall survival of patients with HCC, suggesting their potential use as biomarkers for HCC [105]. The interactions between MMPs and TIMPs have been modeled according to evolutionary game theory, providing a better understanding of their dynamics in the presence or absence of cancer cells, while also offering alternate courses in cancer progression control; restoring the MMP-TIMP balance may represent an important adjuvant therapy, limiting cancer invasion and modulating the metabolisms and interactions between cancer cells and their opponents [106].

## 7. Conclusions

The microenvironment of HCC cells determines the invasiveness and metastasis of tumor cells. A key factor in limiting the aggressiveness of HCC, the ECM integrity, is maintained, among other factors, by normal ratios of MMPs and TIMPs. Overexpression of various MMPs can lead to extreme ECM breakdown and significantly increased EMT. Moreover, MMPs can cross-activate, and the imbalance between MMPs and TIMPs seems to play a major role in cell migration.

## Figures and Tables

**Table 1:** Summary of the roles of MMPs involved in the EMT of HCC and their interactions.

MMP	Category	Role in the EMT of HCC	Cross-activated by MMP	References
MMP-1	Collagenase	Invasion and metastasis	MMP-3, MMP-7, and MMP-10	[21, 25, 43, 44]
MMP-2	Gelatinase	Invasion and metastasis	MMP-1, MMP-7, MMP-12, MMP-13, MMP-14, MMP-15, MMP-16, MMP-17, MMP-24, and MMP-25	[22–26, 44–46]
MMP-3	Stromelysin	Invasion and metastasis	MMP-12	[47–60, 74]
MMP-7	Matrilysin	Invasion and metastasis	MMP-3, MMP-10	[21, 50, 51]
MMP-8	Collagenase	Angiogenesis and migration	MMP-3, MMP-10	[21, 52–54]
MMP-9	Gelatinase	Angiogenesis, invasion, and metastasis	MMP-2, MMP-3, MMP-7, MMP-10, and MMP-13	[21, 24, 55–58, 74]
MMP-10	Stromelysin	Angiogenesis, invasion, and metastasis	N/A	[59–62]
MMP-11	Stromelysin	Invasion and metastasis	N/A	[63, 64]
MMP-13	Collagenase	Invasion and metastasis	MMP-2, MMP-3, MMP-7, MMP-10, MMP-14, and MMP-15	[22, 25, 26, 65, 66]
MMP-14	Membrane-type	Invasion and metastasis	MMP-14	[22, 67, 68]
MMP-16	Membrane-type	Invasion and metastasis	N/A	[40, 69]
MMP-26	Matrilysin	Invasion and metastasis	MMP-26	[25, 70, 71]
MMP-28	Other	Invasion and metastasis	N/A	[72, 73]
